# Defining natural factors that stimulate and inhibit cellulose:xyloglucan hetero‐transglucosylation

**DOI:** 10.1111/tpj.15131

**Published:** 2021-01-21

**Authors:** Klaus Herburger, Lenka Franková, Martina Pičmanová, Anzhou Xin, Frank Meulewaeter, Andrew Hudson, Stephen C. Fry

**Affiliations:** ^1^ The Edinburgh Cell Wall Group Institute of Molecular Plant Sciences School of Biological Sciences The University of Edinburgh Edinburgh EH9 3BF UK; ^2^ BBCC Innovation Center Gent – Trait Research BASF Gent (Zwijnaarde) 9052 Belgium; ^3^ Present address: Section for Plant Glycobiology Department of Plant and Environmental Sciences University of Copenhagen Frederiksberg 1871 Denmark

**Keywords:** cell wall, cellulose, *Equisetum fluviatile*, expansins, hemicelluloses, transglycosylation, xyloglucan

## Abstract

Certain transglucanases can covalently graft cellulose and mixed‐linkage β‐glucan (MLG) as donor substrates onto xyloglucan as acceptor substrate and thus exhibit cellulose:xyloglucan endotransglucosylase (CXE) and MLG:xyloglucan endotransglucosylase (MXE) activities *in vivo* and *in vitro*. However, missing information on factors that stimulate or inhibit these hetero‐transglucosylation reactions limits our insight into their biological functions. To explore factors that influence hetero‐transglucosylation, we studied *Equisetum fluviatile* hetero‐trans‐β‐glucanase (*Ef*HTG), which exhibits both CXE and MXE activity, exceeding its xyloglucan:xyloglucan homo‐transglucosylation (XET) activity. Enzyme assays employed radiolabelled and fluorescently labelled oligomeric acceptor substrates, and were conducted *in vitro* and in cell walls (*in situ*). With whole denatured *Equisetum* cell walls as donor substrate, exogenous *Ef*HTG (extracted from *Equisetum* or produced in *Pichia*) exhibited all three activities (CXE, MXE, XET) in competition with each other. Acting on pure cellulose as donor substrate, the CXE action of *Pichia*‐produced *Ef*HTG was up to approximately 300% increased by addition of methanol‐boiled *Equisetum* extracts; there was no similar effect when the same enzyme acted on soluble donors (MLG or xyloglucan). The methanol‐stable factor is proposed to be expansin‐like, a suggestion supported by observations of pH dependence. Screening numerous low‐molecular‐weight compounds for hetero‐transglucanase inhibition showed that cellobiose was highly effective, inhibiting the abundant endogenous CXE and MXE (but not XET) action in *Equisetum* internodes. Furthermore, cellobiose retarded *Equisetum* stem elongation, potentially owing to its effect on hetero‐transglucosylation reactions. This work provides insight and tools to further study the role of cellulose hetero‐transglucosylation *in planta* by identifying factors that govern this reaction.

## Significance Statement

The enzyme HTG can graft segments of cellulose molecules onto xyloglucan (a hemicellulose), thereby re‐structuring the cell wall via hetero‐transglycosylation. In native walls, two endogenous hemicelluloses competed with cellulose (and with each other) as substrate. HTG more readily selected cellulose as substrate if an expansin‐enriched preparation was added. Hetero‐transglycosylation was inhibited by cellobiose – a potential tool for exploring HTG’s biological functions. Interestingly, cellobiose retarded *Equisetum* stem elongation, suggesting a role for HTG in growth.

## Graphical abstract

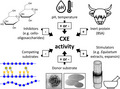

## INTRODUCTION

Plant cells are surrounded by complex polysaccharide‐rich cell walls, which determine plant morphology and play a crucial role during development (Popper *et al*., 2011). Cell wall polysaccharides fall into three classes: cellulose, hemicellulose or pectin (Harholt *et al*., 2010; Scheller and Ulvskov, 2010). Cellulose molecules comprise up to 10 000 β‐(1→4)‐linked glucose monomers and these unbranched chains, synthesised at the plasma membrane, aggregate into fibrils (Klemm *et al*., 2005). Pectin is a complex anionic polysaccharide rich in galacturonic acid residues (Albersheim *et al*., 2010). Major land‐plant hemicelluloses consist of a β‐(1→4)‐linked glucose and/or mannose or xylose backbone and some possess side chains: for example, xyloglucan (XyG), the most abundant hemicellulose in most land‐plant primary walls (Popper *et al*., 2011), carries α‐(1→6)‐linked xylose side chains with or without attached galactose and fucose. These three polysaccharide classes are considered to form dense networks (Park and Cosgrove, 2012).

During many physiological processes and whenever plants exercise morphological changes, their cell walls require modifications (Cosgrove, 2015; Höfte and Voxeur, 2017). A major enzyme class involved in modifying polysaccharides after secretion into the wall is the transglucanases belonging to the glycoside hydrolase family 16 (GH16; Behar *et al*., 2018). The most intensively studied are XyG endotransglucosylase/hydrolases (XTHs; EC 2.4.1.207; Fry *et al*., 1992; Eklöf and Brumer, 2010; Franková and Fry, 2020), which form XyG:XyG homo‐polymers by cutting a XyG molecule and grafting it onto another XyG nearby (XyG:XyG endotransglucosylase (XET) activity). Land‐plant genomes typically encode more than 30 XTHs and some of these have been shown to be involved in numerous physiological and developmental processes (for a summary of the enzymology and biological roles of XTHs see Franková and Fry (2013)). Other homo‐transglycanase activities reported in extracts prepared from plants include trans‐β‐xylanase (Franková and Fry, 2011, 2020) and trans‐β‐mannanase (Schröder *et al*., 2004). (We have used ‘gluc...’ when we wish to specify that a glucosyl linkage is being cleaved and ‘glyc...’ when we do not wish to specify the type of sugar.)

On the other hand, recent studies report certain hetero‐transglucanase activities that can catalyse the cleavage of cellulose chains or soluble cellulose derivatives, followed by their covalent attachment to XyG oligosaccharides (XGOs; Hrmova *et al*., 2007; Simmons *et al*., 2015; Shinohara *et al*., 2017; Stratilová *et al*., 2019; Herburger *et al*., 2020a). While all these studies provide detailed descriptions of the enzymology of the tested transglucanases, they do not explore factors which may stimulate or inhibit hetero‐transglucosylation activities. Furthermore, in most cases, ‘cellulose’ hetero‐transglucosylation activities were tested on artificial soluble cellulosic substrates (e.g. cellulose acetate, hydroxyethylcellulose or phosphoric acid‐treated amorphous cellulose; Maris *et al*., 2011; Shinohara *et al*., 2017) and it is unlikely that such substrates occur in native plant cell walls, where hetero‐transglucanases are active. The only hetero‐transglucanase that exhibits cellulose:XyG endotransglucosylase (CXE) activity – that is, can act on native insoluble plant cellulose (e.g. cotton‐sourced filter paper) – is hetero‐trans‐β‐glucanase (*Ef*HTG), which was discovered recently in the early‐diverging fern ally, *Equisetum* (Simmons *et al*., 2015). Besides CXE activity, *Ef*HTG catalyses two further transglucosylation reactions at high rates: it has mixed‐linkage β‐d‐glucan (MLG):XyG endotransglucosylase (MXE) and conventional XET activity (Fry *et al*., 2008; Mohler et al., 2013; Simmons *et al*., 2015; Simmons and Fry, 2017).

The aim of the present study was to investigate factors that stimulate and inhibit hetero‐transglucosylation by focusing on the well‐characterised hetero‐trans‐β‐glucanase *Ef*HTG. Plants are indeed known to contain factors that modulate XTHs’ activity (Sharples *et al*., 2017; Nguyen‐Phan and Fry, 2019). We thus hypothesised that plants possess components governing CXE and/or MXE activity.

We found that *Equisetum* contains extractable factors that strongly boost CXE but not XET or MXE activity. We also show that a set of cell wall‐related oligosaccharides can be used to selectively inhibit hetero‐transglucanase activities of *Ef*HTG (*in vitro* and *in situ* and potentially *in planta*). This can serve as a solid foundation for future ‘chemical genetics’ studies and will inform the biotechnological use of cellulose hetero‐transglucosylation (Herburger *et al*., 2020b).

## RESULTS

### *Ef*HTG has a high longevity and produces stable cellulose–xyloglucan bonds

*Ef*HTG shows the highest CXE:XET activity ratio of all known hetero‐transglucanases (Hrmova *et al*., 2007; Stratilová *et al*., 2010; Simmons *et al*., 2015; Shinohara *et al*., 2017; Stratilová *et al*., 2019). It also exhibits high MXE activity (Simmons and Fry, 2017). As *Ef*HTG possesses two major hetero‐transglucanase activities, we studied this enzyme to explore hetero‐transglucosylation in numerous *in‐vitro* and in *in‐situ* experiments.

**Figure 1:**
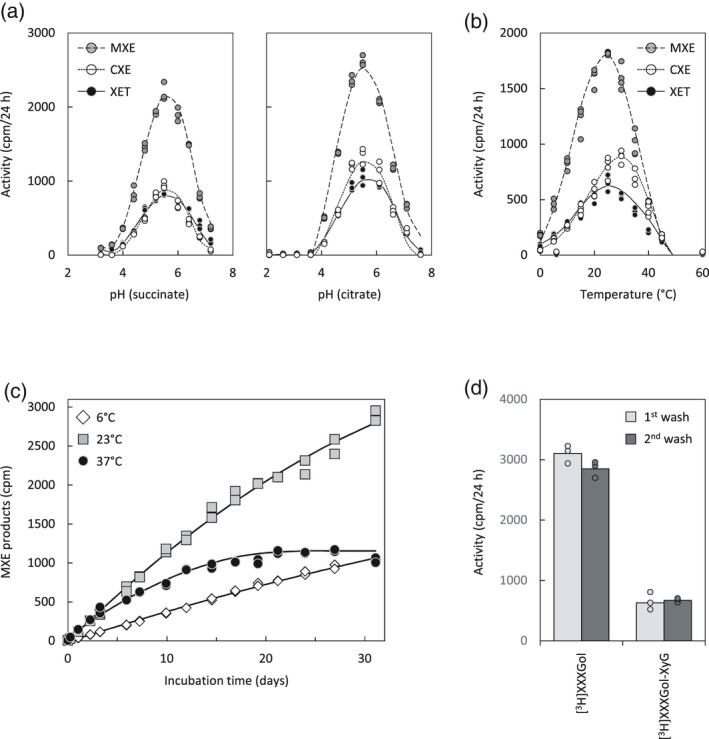
*Ef*HTG activities under varied pH, temperature, incubation time or acceptor substrate length. Activities were tested *in vitro* with XyG (XET), MLG (MXE) and/or NaOH‐pre‐treated filter paper (CXE) as donor and [^3^H]XXXGol or polymeric [^3^H]XyG as acceptor substrate. Enzyme: *Pichia*‐produced *Ef*HTG. (a) pH dependence of *Ef*HTG activities in succinate (left) or citrate buffer (right); *n* = 3; data points are shown as circles. (b) Temperature dependence of *Ef*HTG activities at pH 5.5 after 24 h incubation; *n* = 4; data points are shown as circles. (c) MXE activity measured over 30 days at 6, 23 and 37°C; *n* = 2. (d) Comparing CXE activity with [^3^H]XXXGol or high‐molecular‐weight [^3^H]XyG as acceptor substrates; the cellulosic product was washed twice in 6 m NaOH: wash 1 was done at 20°C for 16 h and wash 2 at 100°C for 1 h; *n* = 3; data points are shown as circles.

To set up optimal conditions for the following enzyme assays, we evaluated the pH and temperature dependence of *Ef*HTG’s activities. XET, MXE and CXE activity exhibited similar pH optima (pH approximately 5.6; Figure 1a, Table S1), close to those of most *Arabidopsis thaliana* XTHs (Purugganan *et al*., 1997; Maris *et al*., 2011). Appreciable activities at typical apoplastic pH values (approximately 4.8; Grignon and Sentenac, 1991) suggest that *Ef*HTG can act in plant cell walls. Indeed, all three of its actions (XET, MXE, CXE) have recently been documented in native cell walls (Herburger *et al*., 2020a).

*Ef*HTG was active across a broad temperature range, from 0 to 45°C (Figure 1b). Interestingly, the temperature optima of activities assayed on soluble donor substrates (XET, MXE) were similar (24–25°C), while the temperature optimum for CXE activity was noticeably higher (33.5°C; Table S1). Potentially, immobilisation of HTG on a solid matrix (paper) better protects it from denaturation (Zhang *et al*., 2015). However, all three activities ceased at about 50°C (Figure 1b). Appreciable activity between 5 and 45°C suggests that *Ef*HTG can act during most of the year, in agreement with our finding that *Ef*HTG activities are extractable from field‐grown *Equisetum* during all seasons including winter (Herburger *et al*., 2020a).

Lengthy *in‐vitro* incubations revealed a remarkable longevity of *Ef*HTG, with essentially linear MXE product formation persisting for at least 1 month at 6 or 23°C (Figure 1c). At 37°C, the rate was higher for the first 5 days, followed by a decrease, indicating gradual enzyme denaturation (Figure 1c).

We also confirmed that *Ef*HTG can graft cellulose onto high‐molecular‐weight [^3^H]XyG, albeit at lower rates than onto the oligosaccharide [^3^H]XXXGol (Figure 1d). The cellulose–[^3^H]XyG products were equally insoluble in boiling 6 m NaOH (Figure 1d, ‘2nd wash’).

### *Ef*HTG can simultaneously act on cellulose and tightly bound hemicelluloses

**Figure 2:**
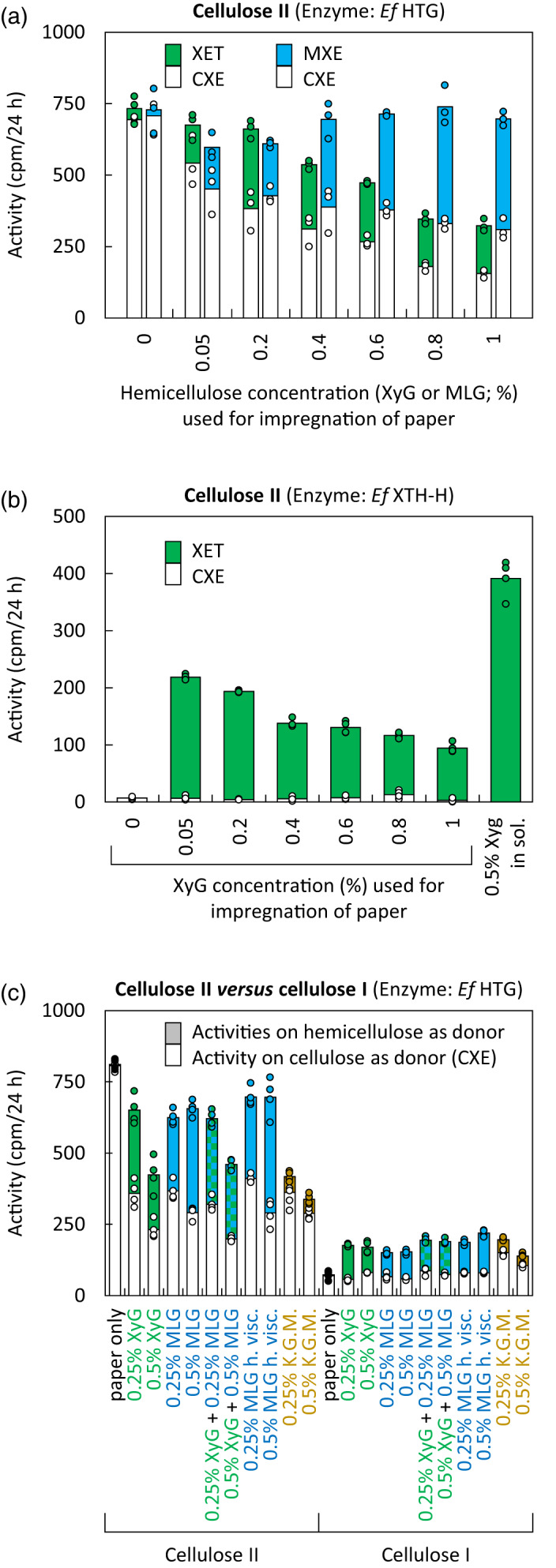
Transglucanase activities of *Ef*HTG and *Ef*XTH‐H in the presence of more than one potential donor substrate. Acceptor substrate: [^3^H]XXXGol. (a) Donor substrates: cellulose II (alkali‐pre‐treated filter paper) impregnated with solutions of increasing XyG or MLG concentration and then washed in water before quantification of transglucanase activities (CXE and XET or CXE and MXE). Enzyme: *Pichia*‐produced *Ef*HTG; *n* = 3; data points are shown as circles. (b) Corresponding assays with *Pichia*‐produced *Ef*XTH‐H acting on cellulose II impregnated with XyG and water‐washed; *n* = 4; data points are shown as circles. (c) As (a) but donor substrates: cellulose I and II (untreated and NaOH‐pre‐treated filter papers, respectively) impregnated with different hemicelluloses; *n* = 4; data points are shown as circles. KGM, Konjac glucomannan; MLG h. visc. and l. visc, mixed‐linkage β‐glucan with high or low viscosity.

In the cell wall *in vivo*, transglycanases may have access to a number of polysaccharides, any of which could potentially serve as competing donor substrates. To study such competition *in vitro*, we quantified CXE activity in the presence and absence of other potential donor substrates, using cotton cellulose I or cellulose II (i.e. untreated or alkali‐pre‐treated filter paper, respectively) impregnated with XyG, MLG or konjac glucomannan (KGM) (Figure 2). The hemicellulose‐impregnated papers were washed in water before the addition of a *Pichia*‐produced enzyme (*Ef*HTG or *Ef*XTH‐H) plus [^3^H]XXXGol (XGO acceptor substrate) for transglucanase assays. While the water holding capacity of untreated or alkali‐pre‐treated filter paper was similar (approximately twice their dry weights), water washing removed 59 ± 5% of XyG and 46 ± 5% of MLG from untreated papers but only 39 ± 9% of XyG and 28 ± 6% of MLG from alkali‐pre‐treated filter papers (mean ± SD; *n* = 20). The removed XyG and MLG most likely represent loosely bound donor substrates.

Infiltrating a 1% (w/v) solution of XyG into filter‐paper cellulose (followed by washing away loosely bound XyG) strongly decreased *Ef*HTG’s detectable CXE activity (approximately 78% inhibition of formation of alkali‐insoluble, ^3^H‐labelled polymeric products; Figure 2(a), white bars beneath green bars). Two factors are likely to contribute to this effect: (i) the enzyme’s ability to graft cellulose onto the impregnating XyG molecules (Figure 1d), producing non‐radioactive products (cellulose–XyG) in competition with cellulose‐to‐[^3^H]XGO grafting, which produces radiochemically detectable products; and (b) the enzyme’s ability to catalyse XyG‐to‐[^3^H]XGO transglycosylation (XET activity) in competition with cellulose‐to‐[^3^H]XGO transglycosylation (CXE activity). To distinguish (a) and (b) as factors affecting detectable CXE activity, we can consider the detectable XET reaction rates occurring in the same assays: as expected, negligible ‘XET’ activity was detected on non‐impregnated paper (Figure 2a, green bars), whereas infiltration with 0.2% XyG enabled maximal detectable XET activity (XyG‐to‐[^3^H]XGO transglycosylation); compared with this rate, infiltrating a higher concentration of XyG (1% w/v) diminished the detectable XET rate by only approximately 40%. Of factors (a) and (b) above, only (a) is relevant in the case of measured XET activity – *viz*. undetectable polymer‐to‐XyG transglycosylation competing with detectable polymer‐to‐[^3^H]XGO transglycosylation. The difference between the effect of 1% XyG impregnation on CXE and that on XET (78% versus 40% inhibition) suggests that the main competitive factor operating in the CXE assays was (b) – the ability of XyG to compete with cellulose as donor substrate.

*Ef*XTH‐H does not exhibit appreciable CXE activity (Holland *et al*., 2020; Figure S1a), but it was able to catalyse detectable XyG‐to‐[^3^H]XGO (XET) transglycosylation in XyG‐impregnated paper (Figure 2b). The highest XET activity thus detected was on papers that had been infiltrated with 0.05% (w/v) XyG (and, as above, washed free of loosely bound XyG). Increasing to 1% (w/v) the concentration of the XyG solution used for infiltration caused a 57% inhibition of detected reactions. This is reasonably close to the 40% inhibition of detectable XET reactions observed with *Ef*HTG (Figure 2a) and is explained by undetectable XyG‐to‐XyG reactions competing with detectable XyG‐to‐[^3^H]XGO reactions. The preferred donor substrate of *Ef*XTH‐H was soluble XyG rather than paper‐bound XyG (Figure 2b), suggesting that hydrogen‐bonding to cellulose renders XyG a somewhat less effective donor substrate.

Impregnating cellulose II papers with MLG, as with XyG, diminished detectable CXE activity (Figure 2a; white bars beneath blue bars); the biggest effect was 56% inhibition, caused by impregnation with 1% (w/v) MLG. This is a smaller effect than the 78% inhibition seen with XyG impregnation, and is assumed to reflect only factor (b) above – hemicellulose‐to‐[^3^H]XGO transglycosylation reactions competing with cellulose‐to‐[^3^H]XGO reactions. Indeed, *Pichia*‐produced *Ef*HTG prefers MLG over cellulose as a donor substrate under these assay conditions (e.g. Figure 1a). The equivalent to factor (a) proposed above (i.e. cellulose‐to‐hemicellulose transglycosylation competing with cellulose‐to‐[^3^H]XGO transglycosylation) would not apply in the case of MLG impregnation because *Ef*HTG cannot use MLG as an acceptor substrate (Simmons *et al*., 2015). In other words, increasing the concentration of impregnating MLG increases the pool of preferred donor substrates and shifts transglucosylation events from CXE to MXE activity.

Interestingly, impregnating papers with konjac glucomannan (KGM), which is only a poor transglucanase substrate (Herburger *et al*., 2020b), strongly decreased detectable CXE activity by up to 64% (cf. up to 64% with MLG impregnation and 71% with XyG impregnation) (Figure 2c). Potentially KGM coated the cellulose fibres, decreasing their accessibility to *Ef*HTG. While the present study focused on hemicelluloses and cellulose – the substrates for hetero‐transglucosylation – the presence of other cell wall components might influence transglucanase actions too. Homogalacturonan occurs in *Equisetum* cell walls (Sørensen *et al*., 2008) and can mask hemicelluloses, decreasing their accessibility to proteinaceous cell wall probes (Marcus *et al*., 2008) and potentially to cell wall‐remodelling enzymes.

We also tested the effect of adding two different hemicellulose donor substrates at the same time (Figure 2c). Impregnating cellulose II with 0.5% XyG alone, 0.5% MLG alone or 0.5% XyG plus 0.5% MLG resulted in 71%, 64% or 75% inhibition of detectable CXE activity, respectively. Thus there was only slight synergy between these two hemicelluloses in their ability to compete with cellulose as donor substrate, confirming that even in presence of both competing donor substrates (XyG and MLG) appreciable CXE activity occurs. Impregnating XyG (with or without MLG) did diminish radiochemically detectable XET activity, as reported and interpreted above (Figure 2a).

The above results were obtained with hemicellulose‐impregnated cellulose II (i.e. alkali‐pre‐treated paper). For comparison, we also investigated the native form of cotton cellulose (allomorph I, i.e. alkali‐untreated filter paper). As expected (Simmons *et al*., 2015; Herburger *et al*., 2020a), the measured CXE activity on pure cellulose I was only approximately 9% of that on pure cellulose II (Figure 2c), confirming that alkali‐pre‐treatment increases the accessibility of cellulose to *Ef*HTG. Infiltrating hemicelluloses into cellulose I (followed by washing away the loosely bound hemicelluloses) had little if any effect on measured CXE activity. This may be due to a diminished ability of hemicelluloses to firmly bind to the surfaces of cellulose I as we found for our washing experiments, as previously demonstrated by an approximately 2.5× lower ability of XyG to bind to cotton fibre cellulose than to alkali‐washed pea cellulose (Hayashi *et al*., 1987). In support of this interpretation, the enzyme’s ability to utilise cellulose I‐bound XyG and/or MLG was only a quarter to a half of that with cellulose II‐bound hemicelluloses (Figure 2c). Furthermore, alkali treatment shrinks the area of filter papers by approximately 20% (Herburger *et al*., 2020b), which – in combination with the higher hemicellulose binding of washed cellulose II paper – increases the amount of XyG and/or MLG per area, giving *Ef*HTG more access to hemicellulose donor substrates.

In conclusion, *Ef*HTG can act on both XyG and MLG that are tightly attached (resistant to water‐washing) to cellulose. At the same time, *Ef*HTG can utilise cellulose as donor substrate even if the cellulose fibres are coated by hemicelluloses.

### *Ef*HTG acts on native *Equisetum* cell walls, exhibiting all three activities

**Figure 3:**
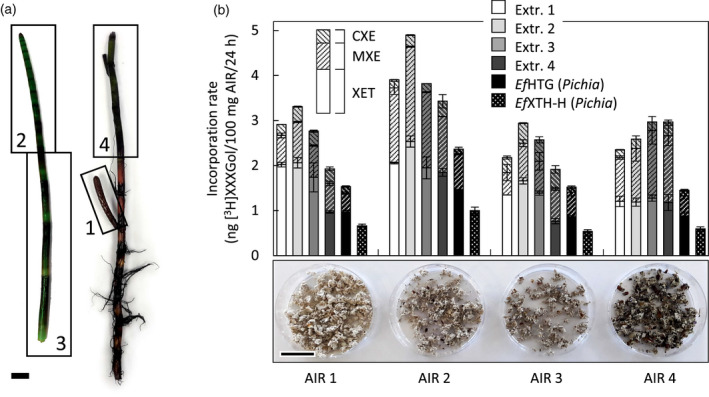
*Ef*HTG acting on native cell walls. (a) *Equisetum* plant, showing zones 1–4, which were used to prepare protein extracts and AIR (scale bar = 20 mm). (b) Three transglucanase activities exhibited by exogenous enzymes applied to *Equisetum* cell walls. Enzymes (see key above histograms): *Ef*HTG and co‐extracted *Ef*XTHs extracted from *Equisetum* zones 1–4 (= Extr. 1–4) or heterologously produced in *Pichia*; the *Pichia*‐produced homo‐transglucanase *Ef*XTH‐H served as a control. Donor substrates (photographs below *x*‐axis; scale bar = 20 mm): *Equisetum* cell wall material (AIR) from organs of different age (zones 1–4 gave AIR 1–4); acceptor substrate: [^3^H]XXXGol; mean ± SD; *n* = 3. XET, MXE and CXE activities are indicated by their ^3^H‐labelled products: XyG‐[^3^H]XXXGol, MLG‐[^3^H]XXXGol and cellulose‐[^3^H]XXXGol, respectively, formed in the same cell walls at the same time.

After confirming that *Ef*HTG transglucanase activities operate at high rates on pure cellulose and on cellulose impregnated with competing hemicellulosic donor substrates, we employed a transglucanase assay that mimics *in‐planta* conditions. The donor substrates were ethanol‐denatured cell walls (alcohol‐insoluble residue (AIR); Figure 3b photographs) from *Equisetum* shoots at different developmental stages (Figure 3a), the enzyme solutions were either total extracts prepared from the same shoots or *Pichia*‐produced transglucanases, and the acceptor substrate was [^3^H]XXXGol. Acting on individual pure donor substrates, such *Equisetum* protein extracts and *Pichia*‐produced *Ef*HTG exhibited high MXE:XET activity ratios (1.3–5.3) and CXE:XET ratios (0.8–3.3; Figure 3a; Figure S1a). In contrast, as expected, *Pichia*‐produced *Ef*XTH‐H exhibited almost only XET activity (Figure 3a; Figure S1a).

XET:MXE:CXE ratios were remarkably different when transglucanases acted on *Equisetum* cell walls, which contain all three *Ef*HTG donor substrates (XyG, MLG, cellulose) in naturally relevant concentrations and architecture. Total *Equisetum* extracts and *Pichia*‐produced *Ef*HTG often gave MXE:XET ratios of roughly 1 rather than, as above, MXE predominating. Indeed, when acting on AIR from the youngest shoots (AIR1, Figure 3b), the same enzyme preparations consistently gave MXE:XET ratios less than 1 (0.3–0.6). This may reflect the relatively low MLG abundance in the cell walls of young *Equisetum* shoots (Sørensen *et al*., 2008) (Figure 3b; Figure S1b). Only with extracts from the oldest shoots (i.e. with maximal HTG:XTH) acting on AIR from the oldest shoots (i.e. with maximal MLG:XyG) did MXE activity slightly predominate (MXE:XET ≈ 1.25). CXE activity was rather variable – between approximately 5% and approximately 20% of total transglucanase activity (Figure 3b; Figure S1b). However, *Pichia*‐produced *Ef*HTG exhibited constant relative CXE activities (approximately 4–8% of total transglucanase activity) regardless of the AIR selected. The main conclusion is that both the hetero‐transglucanase activities (MXE and CXE) can operate simultaneously on intact *Equisetum* cell walls.

### Plant extracts contain CXE activity enhancers

**Figure 4:**
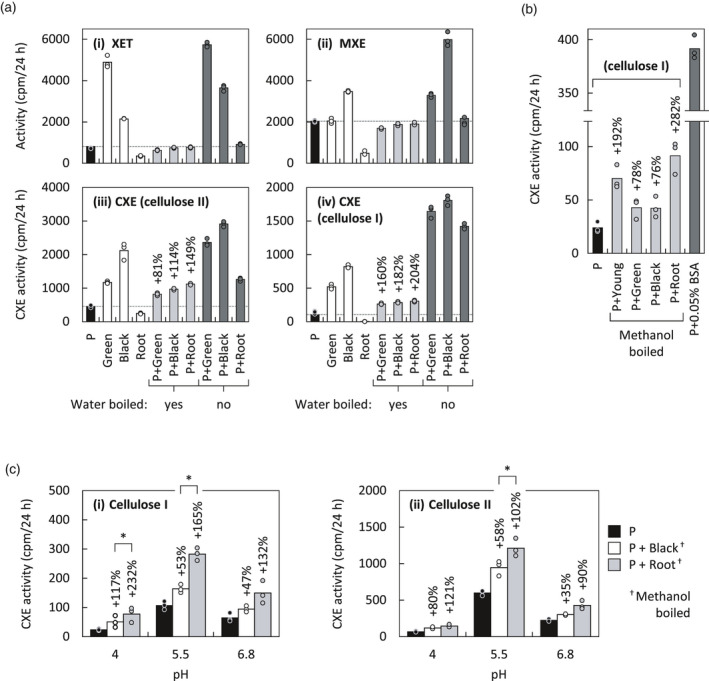
Stimulatory effect of non‐enzymatic *Equisetum* polymers. Acceptor substrate in all cases: [^3^H]XXXGol. (a) Effect of *Equisetum* extracts on activities of *Pichia*‐produced *Ef*HTG. Donor substrates: (i) XyG, (ii) MLG, (iii) cellulose II, (iv) cellulose I, for detection of XET, MXE, CXE and CXE activities, respectively. Enzymes (*x*‐axis): Green, from green shoot middle; Black, from blackish shoot base; Root, from roots; P, *Pichia*‐produced *Ef*HTG; and combinations thereof. ‘Boiled’ indicates that the *Equisetum* enzyme extracts (Green, Black, Root), but not the P, had been pre‐incubated in aqueous solution at 100°C for 5 sec, which is expected to denature *Ef*HTG and XTHs but not expansins (Wang *et al*., 2014) or the XTH‐activating factor (XAF) of Nguyen‐Phan and Fry (2019). Stimulating effects of ‘boiled’ *Equisetum* extracts on CXE activities are indicated as a percentage above columns. Dashed lines indicate activities of *Pichia*‐produced *Ef*HTG only; *n* = 3; data points are shown as circles. (b) CXE activity of *Pichia*‐produced *Ef*HTG in the presence of activity‐stimulating *Equisetum* crude extracts that had been boiled in methanol (common procedure to maintain expansins but not enzymes active). For comparison, CXE activity on NaOH‐pre‐treated paper and in presence of 0.05% BSA is shown (dark grey column); *n* = 3; data points are shown as circles. Designation of extracts is as in (a), plus ‘Young’ = from green shot top. (c) Same experiment as in (b), but performed at three different pH values and in presence of 3% (w/v) BSA. Methanol‐boiled extracts from the blackish shoot base and roots were used. Statistically significant differences between CXE activity stimulations by extracts from roots or blackish shoot bases (determined by standard *t*‐tests) are indicated by asterisks; *n* = 3; data points are shown as circles, *P* < 0.05. Cellulose I, plain Whatman No. 1 paper; cellulose II, alkali‐treated Whatman No. 1 paper.

The above data show that differently aged *Equisetum* cell wall material being acted on by protein extracts from differently aged *Equisetum* shoots produced different amounts and ratios of XET, MXE and CXE products. This suggests that the cell wall composition (e.g. polysaccharide ratios) and architecture (e.g. accessibility to enzymes), and/or the presence of co‐extracted substances (e.g. transglucanase‐inhibiting or ‐stimulating factors; Nguyen‐Phan and Fry, 2019), strongly affect transglucanase activities. In the following, we explored the ‘co‐extracted substance’ hypothesis. We added *Equisetum* extracts to *Pichia*‐produced *Ef*HTG and quantified its activities (Figure 4a). The addition of denatured *Equisetum* extracts (inactivated in boiling water) had negligible effect on the XET and MXE activities of *Pichia*‐produced *Ef*HTG (Figure 4a; graphs i and ii, pale grey bars), although controls showed that the same *Equisetum* extracts when not boiled contributed high XET and MXE activities as expected (Figure 4a; graphs i and ii, white and dark grey bars). However, the addition of heat‐denatured *Equisetum* extracts strongly increased the CXE activity of *Pichia*‐produced *Ef*HTG (Figure 4a; graphs iii and iv, pale grey bars), especially when natural cellulose I was the donor substrate (Figure 4a; graph iv): for example, adding denatured root extracts approximately trebled the CXE activity (Figure 4a; graph iv). The short treatment in boiling water (approximately 5 sec) was clearly sufficient to inactivate enzymes such as HTG and XTHs (Figure 4a; graphs i and ii), but may not have inactivated co‐extracted expansins, whose activity has been reported to withstand boiling in methanol (approximately 65°C; McQueen‐Mason *et al*., 1992; Fry, 1994; Wang *et al*., 2020). A bacterial and a fungal expansin even kept more than two‐thirds of their activities after water‐boiling for 5 min (Wang *et al*., 2014). Indeed, adding *Equisetum* extracts that had been boiled in methanol and dialysed still strongly increased the CXE activity of *Pichia*‐produced *Ef*HTG up to 3.8‐fold (Figure 4b) and this may reflect an effect of co‐extracted α‐ and/or β‐expansins.

**Figure 5:**
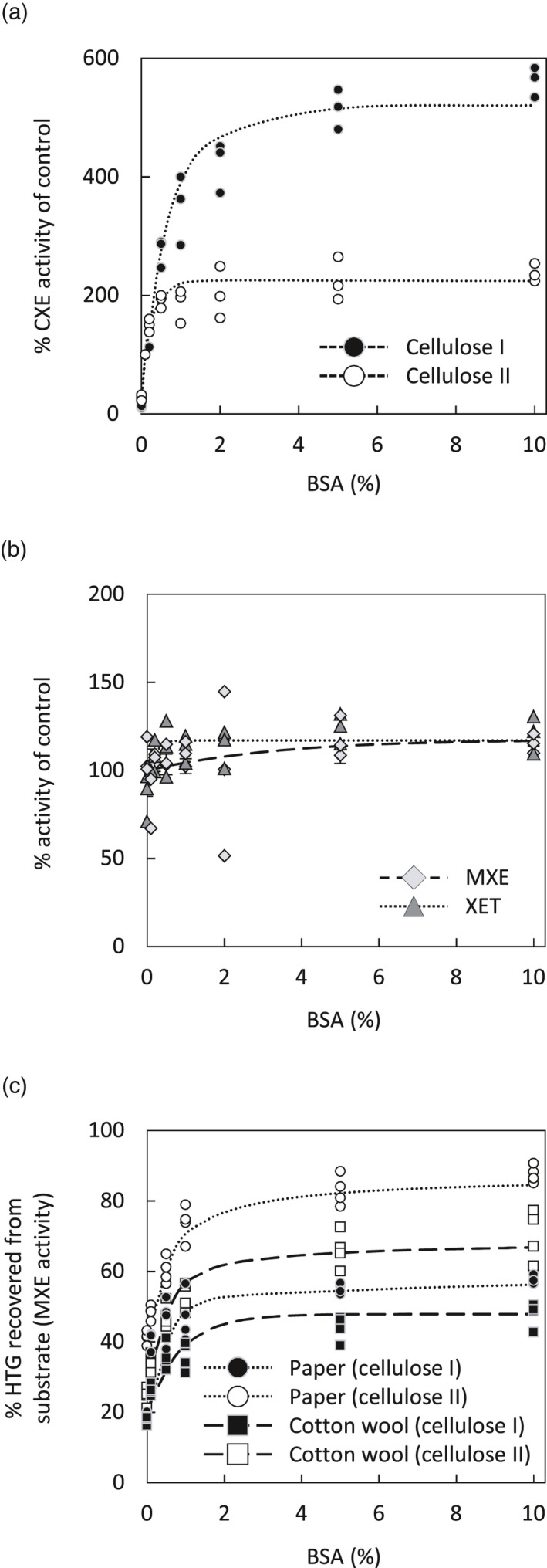
Effect of BSA on *Ef*HTG activities (XET, MXE, CXE). (a) Effect of increasing BSA concentrations on CXE activity expressed as a percentage of the activity obtained in the presence of the previously used routing BSA concentration (0.1% (w/v)). Donor substrate: untreated or NaOH‐pre‐treated filter paper. Acceptor substrate: [^3^H]XXXGol. Enzyme: *Pichia*‐produced *Ef*HTG. *n* = 3; data points are shown as circles. (b) Analogous to the experiment in (a), but testing the effect of increasing BSA concentrations on XET and MXE activities with soluble donor substrates; *n* = 3; data points are shown as rhombi (MXE) and triangles (XET). (c) Recovery of *Ef*HTG bound to cellulose I and II of paper or cotton wool. *Pichia*‐produced *Ef*HTG was applied onto cellulose (untreated or NaOH‐pre‐treated) in presence of increasing BSA concentrations (0–10%), which was incubated under humid conditions with no acceptor substrate; after 16 h, the cellulose was then washed in buffer to release unbound *Ef*HTG, which was then assayed for its MXE activity with [^3^H]XXXGol as acceptor substrate. The proportion of *Ef*HTG (%) recovered in the presence of increasing BSA concentrations is expressed as solubilised MXE activity; 100% MXE activity was produced by *Ef*HTG which had not been applied onto paper; *n* = 3; data points are shown as circles (paper) and squares (cotton wool). Paper, Whatman No. 1 filter paper; cellulose I, plain paper or cotton wool; cellulose II, alkali‐pre‐treated paper or cotton wool.

The assays in Figure 4(a,b) were performed at the optimum pH of HTG (pH approximately 5.5). At lower pH values, HTG’s CXE activity is strongly reduced (Figure 1a). To determine stimulating effects of methanol‐boiled *Equisetum* extracts on the CXE activity of *Ef*HTG at pH 4 – the optimum of most plant expansins (Choi *et al*., 2008) – we first required to boost CXE activity to appreciable rates at low pH. We found that CXE activity is strongly stimulated by increasing the bovine serum albumin (BSA) concentration (Figure 5a; Figure S2a), which has only minor effects on XET and MXE activity (Figure 5b; Figure S2b). BSA is known to exhibit a surfactant effect, which for example also promotes degradation of cellulose by cellulase (Yang and Wyman, 2006). The positive effect exerted on CXE activity might be caused by BSA preventing HTG sticking strongly to cellulose substrates (paper or cotton wool; Figure 5c) and thus allowing it to act on a larger pool of cellulose chains by diffusing within the filter paper.

The pH optima of CXE and expansin activities are approximately 5.5 (Figure 1a) and approximately 4.0 (McQueen‐Mason *et al*., 1992), respectively. It was therefore of interest to test the effect of methanol‐boiled *Equisetum* extracts on CXE activity at a range of pH values spanning both proteins’ optima. To obtain measureable CXE activity at pH values as low as 4.0, especially on cellulose I, we increased the BSA concentration in assays from 0.1% (Simmons *et al*., 2015) to 3%, which strongly promoted CXE activity (Figure 5a). In accordance with the results in Figure 4(b), addition of methanol‐boiled *Equisetum* extracts stimulated CXE activity most strongly (by up to 3.3‐fold) at pH 4 (Figure 4c; graph i), the pH optimum of expansins. Stimulation was significantly lower (*P* < 0.05) yet still considerable at pH 5.5 and pH 6.8 (2.6‐ and 2.3‐fold, respectively; Figure 4c; graph i; Figure S3). Acting on cellulose II, the stimulation of CXE activity by methanol‐boiled *Equisetum* extracts was lower than on cellulose I, but still considerable (Figure 4c; graph ii). A significantly lower (*P* < 0.05) stimulation of CXE activity by extracts occurred at pH 6.8 (Figure S3). For both celluloses I and II, methanol‐boiled extracts from *Equisetum* root were more effective (some of which significantly (*P* < 0.05)) than those from blackish stem tissue (Figure 4c).

These experiments show that *Equisetum* extracts contain substances that strongly stimulate CXE but not MXE or XET activity. This boost of CXE activity is not only due to a surfactant effect provided by total *Equisetum* proteins, because it even occurs in assays containing high BSA (3% (w/v)). This and the observation that stimulation is strongest on cellulose I and at low pH agrees with recent results showing that bacterial expansin strongly augments cellulose hetero‐transglucosylation (Herburger *et al*., 2020a).

### Cello‐oligosaccharides inhibit HTG‐specific activities

**Figure 6:**
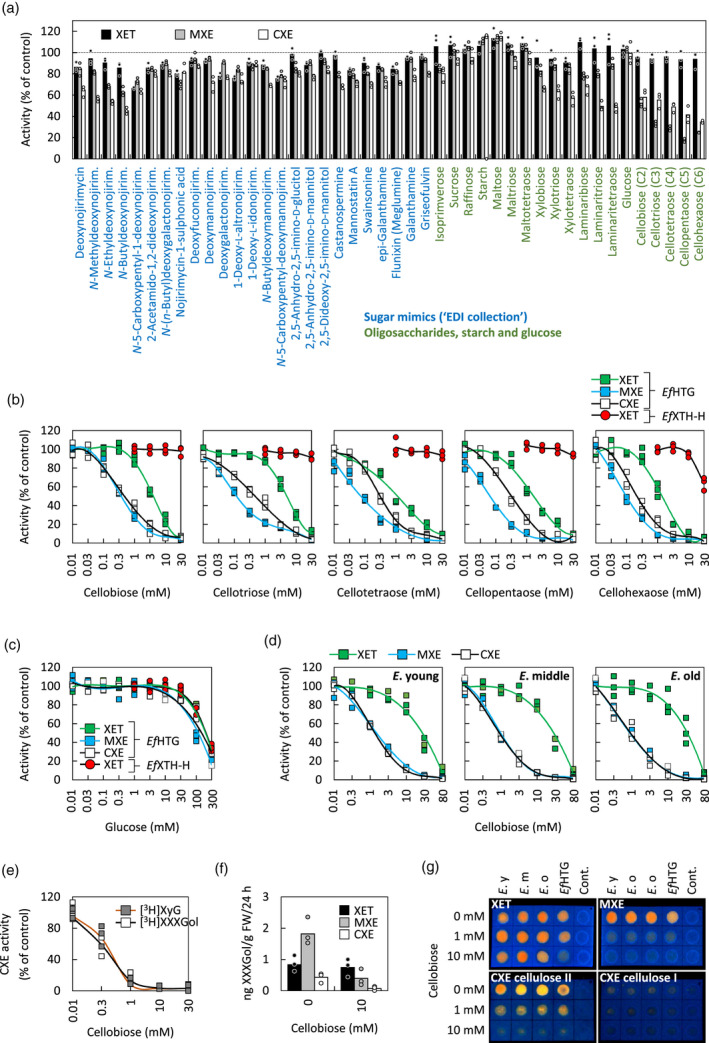
Inhibition of the transglucanase activities of HTG by sugar mimics and oligosaccharides. (a) Inhibitory effect of 25 selected compounds from the EDI xenobiotics collection (Chormova *et al*., 2015), 17 oligosaccharides and glucose (all at 0.3 mm) and starch at 0.2 mg ml^−1^ on the three transglucanase activities of *Pichia*‐produced HTG (acceptor substrate: [^3^H]XXXGol); *n* = 3; controls, *n* = 6. (b) Concentration‐dependent inhibition by cello‐oligosaccharides of the three transglucanase activities of *Pichia*‐produced *Ef*HTG or the XET activity of EfXTH‐H (acceptor: [^3^H]XXXGol); *n* = 3; data points are shown as squares (*Ef*HTG) and circles (*Ef*XTH‐H); controls, *n* = 6. (c) Effect of glucose on the three transglucanase activities of *Pichia*‐produced *Ef*HTG and the XET activity of *Ef*XTH‐H ; *n* = 3; data points are shown as squares (*Ef*HTG) and circles (*Ef*XTH‐H). (d) Concentration‐dependent inhibition by cellobiose of the three transglucanase activities of *Equisetum fluviatile* proteins extracted from young (*E*. young), middle‐aged (*E*. middle) and old (*E*. old) tissues; *n* = 3. (e) Inhibition of CXE activity of *Pichia*‐produced *Ef*HTG (*P*.) by cellobiose (acceptor substrates: [^3^H]XXXGol or [^3^H]XyG); *n* = 3. (f) Inhibitory effect of 10 mM cellobiose on XET, MXE and CXE action (*in situ*) in old *Equisetum* tissue (*E*. old) fed with [^3^H]XXXGol for 24 h (acceptor, [^3^H]XXXGol; enzyme, endogenous); *n* = 3; data points are shown as circles. (g) Dot‐blot showing the inhibitory effects of cellobiose (0–10 mM) on the three transglucanase activities of *Pichia*‐produced *Ef*HTG (*P*.) and *Equisetum* protein extracts from young (*E*. y), middle‐aged (*E*. m) and old (*E*. o) tissues (acceptor: XXXGol‐sulphorhodamine); the cellulose used as donor substrate for the CXE assays was plain paper (cellulose I) or alkali‐pre‐treated paper (cellulose II); the donor substrates for XET and MXE activities were XyG‐ and MLG‐impregnated (NaOH‐untreated) paper, respectively; cont. = no enzyme.

After finding factors that stimulate the CXE activity of HTG, we next performed an inhibitor screen, aiming to find substances that inhibit *Ef*HTG‐specific activities (MXE and CXE, which most transglucanases do not appreciably possess) without affecting XET activity. Such an inhibitor would allow us to block *Ef*HTG‐catalysed hetero‐transglucosylation and thus study its potential functions *in planta*. A set of sugar mimics from the EDI collection (Chormova *et al*., 2015), known to inhibit certain glycohydrolases (Andriotis *et al*., 2016), and various oligosaccharides were tested for inhibitory effects on the MXE, XET and CXE activities (Figure 6). Most sugar mimics at 0.3 mm inhibited all three activities of *Pichia*‐produced *Ef*HTG, though often CXE was affected most: approximately 50% inhibition in the case of *N*‐butyldeoxynojirimycin (Figure 6a). Cello‐, laminari‐ and xylo‐oligosaccharides at 0.3 mm strongly inhibited the CXE and MXE but not XET activities of *Ef*HTG, while glucose showed no effect (Figure 6a). Within each homologous series, larger oligosaccharides were always more effective (Figure 6a). Increasing the concentration of cello‐oligosaccharides to 10 mm very strongly (88–96%) inhibited MXE and CXE activity and inhibited XET activity of *Ef*HTG by 70–90% (Figure 6c). In contrast, 10 or 30 mm glucose showed no inhibitory effect on *Ef*HTG (Figure 6c). Cellobiose inhibited *Ef*HTG’s CXE activity equally whether [^3^H]XXXGol or polymeric [^3^H]XyG was the acceptor (Figure 6e). In contrast, the XET activity of *Pichia*‐produced *Equisetum* homo‐transglucanase *Ef*XTH‐H was unaffected by 10 mm cello‐oligosaccharides (Figure 6b).

Cellobiose effects were further tested on *in‐vitro* activities in *Equisetum* extracts containing native *Ef*HTG plus XTHs (Figure 6d): 10 mm cellobiose inhibited both MXE and CXE activities almost completely, while XET activity remained high (approximately 20% inhibited). Cellobiose at 80 mm almost completely inhibited all three activities (Figure 6d). Radiochemical assays were complemented by dot‐blot assays using XGO‐SR as acceptor, with similar results: 10 mm cellobiose strongly inhibited MXE and CXE but not XET activities of *Equisetum* extracts (Figure 6g). In contrast, 10 mm cellobiose inhibited all three activities of *Pichia*‐produced *Ef*HTG (Figure 6g). In agreement with the *in‐vitro* activity assays, 10 mm cellobiose inhibited MXE and CXE action assayed *in situ* by >80%, while XET action was much less affected (approximately 10% inhibition; Figure 6f).

**Figure 7:**
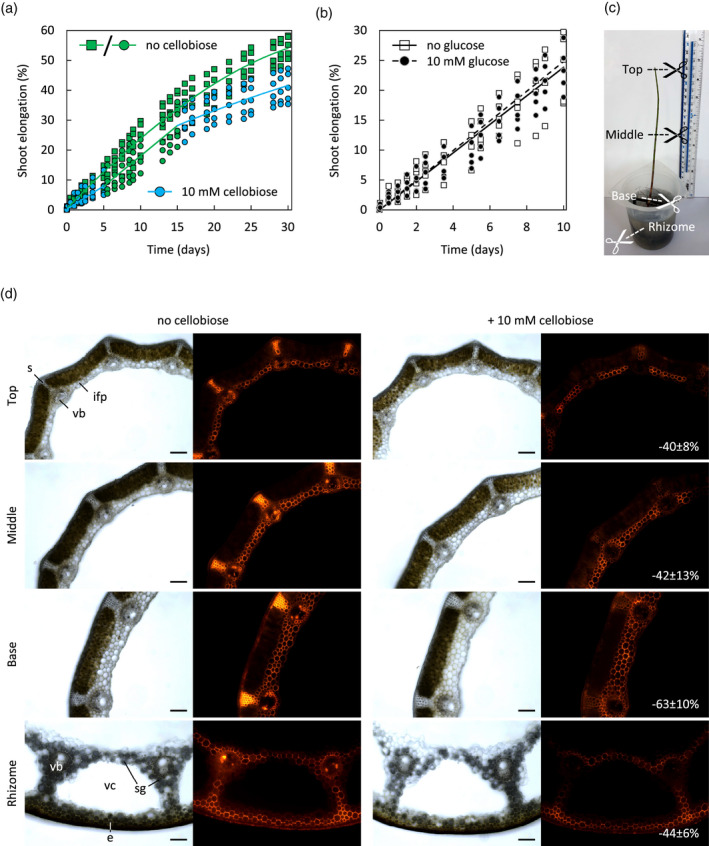
Effect of cellobiose on *Equisetum fluviatile* shoot elongation and *in‐situ* localisation of transglucanase actions. (a) Linear elongation of shoots in the absence of cellobiose (30 days) or in the presence of 10 mm cellobiose for 5 days followed by 10 days without cellobiose and then another 15 days with 10 mm cellobiose; *n* = 5; data points are shown as green squares/circles (no cellobiose) and blue circles (10 mm cellobiose). (b) Linear elongation of shoots (as in (a)) exposed to 10 mm glucose for 10 days does not differ from shoots grown without glucose supplementation. *n* = 5; data points are shown as squares (no glucose) and circles (10 mm glucose). (c) A 4–5‐week *Equisetum fluviatile* shoot connected to a rhizome segment, as used for localisation studies (sites for cross‐sectioning in (d) are marked; green shoot top, middle and base, and non‐green rhizome) and for stem growth measurements in (a). (d) *In‐situ* localisation of exogenous XXXGol‐SR incorporation by endogenous transglucanases acting for 4 h on endogenous donor substrates in sections of *Equisetum* shoots and rhizomes in the absence or presence of 10 mm cellobiose. The measured reduction of fluorescence signal intensity of XXXGol‐SR incorporated during 4 h into total cell walls (cellulose + hemicelluloses) of sections (*in situ*) in the presence of 10 mm cellobiose is shown in % (mean ± SD; *n* = 4). Scale bar = 250 µm.

To determine whether 10 mm cellobiose also inhibits transglucanase action in *Equisetum* tissues *in situ*, we cross‐sectioned top, middle and basal internodes and rhizomes (Figure 7c), and supplied XXXGol‐SR in the presence or absence of 10 mm cellobiose. Fluorescence (Figure 7d) indicates total transglucosylation (XET, MXE and CXE action). Cellobiose decreased the HTG‐generated fluorescence – especially (approximately two‐thirds loss) in basal internodes (Figure 7d), where *Ef*HTG levels exceed levels of EfXTHs (Herburger *et al*., 2020a). In shoot internodes, cellobiose strongly reduced the signal in sclerenchyma and xylem (due predominantly to MXE and CXE action, respectively; Herburger *et al*., 2020a) but not in parenchyma (where XET action predominates; Herburger *et al*., 2020a) (Figure 7d). In rhizomes, which have extended vallecular canals and storage parenchyma, cellobiose reduced fluorescence in all tissues, but strongest in vascular bundles (Figure 7d).

### Inhibition of HTG action retards elongation of *Equisetum* shoots

Since 10 mm cellobiose specifically inhibits hetero‐transglucanase (MXE and CXE) activity and action *in vitro* and *in situ*, while having little effect on XET activity or action (Figures 6 and 7d), we wanted to explore its effect on whole *Equisetum* plants, which exhibit extractable *Ef*HTG activities and actions throughout its stems (Herburger *et al*., 2020a). We hydroponically cultured rapidly elongating *Equisetum* stems, still connected to rhizome segments, in pond water (Figure 7c). Transport of exogenous solutes to the shoot tip was confirmed using safranin O (Figure S4). Addition of 10 mm cellobiose significantly diminished linear stem elongation (0.35 ± 0.11 cm/d; *P* < 0.05; mean ± SD; *n* = 5) relative to the control (0.54 ± 0.13 cm/day; Figure 7a). Removing cellobiose after 5 days restored elongation for the next 10 days (to 0.48 ± 0.13 cm/day; cf. 0.51 ± 0.16 cm/day for controls; Figure 7a), and re‐adding 10 mm cellobiose at 15 days slowed growth again (to 0.21 ± 0.10 cm/day (10 mm cellobiose) and 0.31 ± 0.12 cm/day (no cellobiose); Figure 7a). Glucose (10 mm) did not affect elongation (Figure 7b).

## DISCUSSION

Hetero‐trans‐β‐glucanase (*Ef*HTG) can graft both cellulose and mixed‐linkage glucan onto XyG oligosaccharides, resulting in the formation of very stable hetero‐polymers. We show here that the rate of hetero‐transglucosylation is strongly influenced by many factors, including the presence of stimulating or inhibiting substances, pH, temperature and the nature of the donor substrate (see Graphical Abstract).

### Stimulating cellulose hetero‐transglucosylation

With individual pure donor substrates (XyG, MLG or filter‐paper cellulose), *Pichia*‐produced *Ef*HTG exhibited a CXE:MXE:XET activity ratio of approximately 1:2:1. However, when acting on native *Equisetum* internode cell walls, which contain all three donor substrates, *Ef*HTG’s hetero‐transglucosylation rates were lower, giving CXE:MXE:XET ratios of, for example, approximately 0.2:0.5:1. Interestingly, when native *Equisetum* enzyme extracts acted on the same cell walls, the CXE:MXE:XET activity ratio was approximately 0.4:0.6:1 and thus shifted in favour of CXE activity (Figure 3b). This suggests that *Equisetum* extracts contain components that strongly stimulate cellulose hetero‐transglucosylation.

Expansin augments the activity of cellulose‐active enzymes, an effect which has been intensively studied owing to its potential in enhancing cellulose utilisation during saccharification and other industrial processes (Martinez‐Anaya, 2016). Most studies utilised bacterial expansin, because plant expansins remain difficult to produce heterologously at appreciable yields (Seki *et al*., 2015; Yactayo‐Chang *et al*., 2016). Bacterial expansin can enhance both cellulase activity (Kim *et al*., 2013) and HTG’s CXE activity, but not the latter’s XET and MXE activity (Herburger *et al*., 2020a), suggesting that expansins act on features of (insoluble) cellulose not shared by soluble hemicelluloses. Addition of methanol‐boiled *Equisetum* extracts to CXE assays produced a similar effect on the CXE activity of *Pichia*‐produced *Ef*HTG, and the highest stimulation occurred at the lowest pH tested. Since plant expansins have their pH optimum in the acidic range (Choi *et al*., 2008) and are expressed (at least at the transcriptomic level) throughout *Equisetum* plants (Herburger *et al*., 2020a), it is highly likely that co‐extracted expansins play a role in enhancing CXE activity. As shown before, plants possess XTH‐activating factors (XAFs; cold‐water‐extractable, heat‐stable polymers), which can desorb XTHs from their cell wall binding sites, activating and enabling them to re‐structure XyG *in vivo* (Sharples *et al*., 2017; Nguyen‐Phan and Fry, 2019). BSA had a similar effect on HTG immobilised on filter paper or cotton wool: washing these cellulose preparations in presence of >2% (w/v) BSA solubilised more than twice as much *Ef*HTG than washing with water (Figure 4). However, the effect of *Equisetum* extracts and bacterial expansin on *Ef*HTG is different from that of BSA, because it occurs even in presence of very high BSA and is thus due to interactions with the substrate (cellulose) rather than with the transglucanase itself.

Cellulose hetero‐transglucosylation has great biotechnological potential, allowing covalent incorporation of a commercially valuable ‘cargo’ (attached to an XGO) into cellulosic materials by non‐polluting procedures (Herburger *et al*., 2020b). Advantageously for industrial applications, *Ef*HTG has a remarkable longevity, continuously acting for >1 month at room temperature (Figure 1c) in *in‐vitro* systems, and its performance can be increased inexpensively by addition of *Equisetum* extracts, bacterial expansin and BSA (or potentially any other inert protein). The latter additive would allow recovery of most of the enzyme after use, helping to establish highly efficient production cycles.

Decreasing the incubation temperature from 23 to 6°C caused an approximately 67% decrease in the MXE activity of *Ef*HTG (Figure 1b), indicating a *Q*_10_ of approximately 1.9. Thus HTG is slightly more cold‐tolerant than many enzymes, which typically have a *Q*_10_ of approximately 2. The ability of HTG to continue operating, potentially strengthening structural tissues, in cold conditions may be advantageous in overwintering *Equisetum* organs, for example the submerged *E. fluviatile* stems in a frozen pond.

### Inhibiting cellulose hetero‐transglucosylation

In our search for inhibitors of HTG, we tested a wide range of sugar mimics, many of which have been reported to inhibit various glycosylhydrolases (e.g. Andriotis *et al*., 2016). At 0.3 mm, some of these did partially inhibit HTG, though never more than about 50%. We found that cello‐oligosaccharides (C2–C6) are efficient inhibitors of *Equisetum* HTG, while glucose has no inhibitory effects. MXE and CXE activity – using unbranched donor substrates (MLG and cellulose, respectively) – were affected strongest, while the XET activity of HTG required higher oligosaccharide concentrations for inhibition. However, a comparison of the XET activities of HTG and a standard *Equisetum* homo‐transglucanase (*Ef*XTH‐H), which exhibits negligible MXE and CXE activity, showed that the XET activity of HTG is more susceptible to inhibition than that of XTH‐H. These different inhibitory effects of cellobiose might be explained by 3D modelling, which showed that (i) XyG exhibits three more interactions with HTG’s active site than does cellulose or MLG, but (ii) other standard XTHs still exhibit two more interactions with XyG than does *Ef*HTG (Simmons *et al*., 2015). This suggests that donor XyG binds more strongly to HTG’s active site than does MLG or cellulose and thus is less likely to be affected by cellobiose competition, which would also fit into the donor substrate binding pocket (Simmons *et al*., 2015). Both HTG and conventional XTHs manifest enzyme–substrate interactions with four negative‐ and two positive‐subsite glucoses. However, in conventional XTHs, XyG is stabilised by additional interactions with the –3, +1 and +2 subsite xyloses thus preventing cello‐oligosaccharides functioning as competing acceptor substrate. This helps to explain why only high concentrations of cellohexaose, but not C2–C5, perceptibly inhibited the XET activity of XTH‐H (Figure 6b).

The effectiveness of transglucanase inhibition by cellobiose was in the following order: CXE activity of *Ef*HTG ≈ MXE activity of *Ef*HTG > XET activity of *Ef*HTG >> XET activity of EfXTH‐H (Figure 6b). Therefore cellobiose might be used as a relatively specific inhibitor of the two hetero‐transglycosylation reactions *in planta*. Interestingly, we found that 10 mm cellobiose significantly diminished the elongation of *Equisetum* stems, while glucose (which is not an HTG inhibitor; Figure 6a,e) had no effect. This might corroborate HTG’s role in cell wall mechanics, suggesting that MXE product formation in sclerenchyma, and CXE product formation in tissues around cavities and in vascular bundles (Herburger *et al*., 2020a), is required for high stem elongation rates. Both MXE and CXE action occur abundantly in strengthening tissues throughout *Equisetum* stems (Herburger *et al*., 2020a). In particular, cellulose:XyG junctions might serves as stabilising ‘biomechanical hotspots’ in the cell wall (Wang *et al*., 2013), because their removal enables cell wall creep (Park and Cosgrove, 2012). It is possible that CXE action has its role in continuously helping to re‐establish stabilising cellulose:XyG tethers in expanding tissues, allowing for a modulated growth rate. Further studies might explore the role of hetero‐transglucosylation in cell expansion, for example, by studying whether hetero‐polymer formation correlates with the cell wall expansion rate and direction and with the deposition of new cell wall material.

In general, cellobiose is an advantageous agent for ‘chemical genetics’ studies because it is non‐toxic, highly water‐soluble and membrane‐impermeant – likely to remain largely apoplastic (the location of HTG, the enzyme of interest). In contrast to, for example, cellotriose (Johnson *et al*., 2018), cellobiose does not trigger strong stress responses (e.g. callose deposition or production of reactive oxygen species) in Arabidopsis seedlings without the simultaneous presence of elicitor‐active peptides such as bacterial flagellin (de Azevedo Souza *et al*., 2017). However, cellobiose can upregulate stress‐related WRKY transcription factors, activate MAP‐kinases involved in immune signalling and cause a transient intracellular calcium spike in Arabidopsis that might also be a stress response (de Azevedo Souza *et al*., 2017). Thus, the reduction of *Equisetum* stem elongation might partially reflect a stress response triggered by cellobiose. Arabidopsis seedlings grown with 10 mm cellobiose slightly increased their fresh weight, possibly through the activity of a β‐(1,4)‐hydrolase, which breaks down supplied cellobiose and thus increases the availability of glucose as a carbon source (de Azevedo Souza *et al*., 2017).

However, supplying glucose instead of cellobiose to *Equisetum* did not affect its stem elongation rate (Figure 7b). This suggests that *Equisetum* and Arabidopsis respond differently to glucose/cellobiose exposure and highlights the relevance of studying non‐model organisms to gain a more comprehensive insight into carbohydrate‐mediated plant responses to exogenous stimuli.

In conclusion, this study showed that *Equisetum* tissues contain factors that strongly enhance – more than 2.5‐fold – cellulose hetero‐transglucosylation by *Ef*HTG. Because of the similar pattern of enhancement (highest at low pH, negligible effects on XET and MXE activity), we propose that plant expansins are involved. This provides a solid foundation for further studies, exploring potential roles of synergy between transglucanases and expansins for cell wall formation and remodelling, which govern plant cell expansion and organ strengthening. Furthermore, efforts to introduce cellulose hetero‐transglucanases genetically into other plants for, for example, tissue strengthening could benefit from a simultaneous upregulation of expansin action. Our inhibitor studies show that (hetero‐)transglucanase activities/actions can be inhibited, opening the potential for ‘chemical genetics’ studies. This would be particularly valuable for inhibiting functions of transglucanases, which are difficult to knock out or knock down, for example in non‐model organisms such as *Equisetum* or if excessive genetic redundancy precludes deactivation of all relevant genes. Finally, we suggest that future studies aiming at drawing conclusions from *in‐vitro* experiments – with commercial donor substrates and/or heterologously produced enzyme – on the actual biological roles of transglucanases should consider that numerous factors exist that heavily influence the rate and ratios of transglucosylation activities.

## EXPERIMENTAL PROCEDURES

### Plant sources and materials

*Equisetum fluviatile* was grown in a pond at the Institute of Molecular Plant Sciences of the University of Edinburgh (Edinburgh, UK) or collected from the Pentland Hills (Edinburgh). *Tamarindus indica* seed XyG was from Dainippon Pharmaceutical Co. (Osaka, Japan); barley (*Hordeum vulgare*) MLG (β‐glucan; medium and high viscosity) and lichenase (from *Bacillus subtilis*) were from Megazyme Inc. (Wicklow, Ireland). XXXGol–sulphorhodamine (XXXGol‐SR) was prepared as previously described (Hetherington and Fry, 1993; Miller, *et al*., 2007) and obtained from EDIPOS (http://fry.bio.ed.ac.uk//edipos.html) (the nomenclature of XGOs (e.g. XXXGol) follows Fry *et al*., (1993)). Radiolabelled XyG ([^3^H]XyG) was prepared according to Herburger *et al*. (2020a). Other chemicals were purchased mainly from Sigma‐Aldrich (Poole, UK).

### Heterologous protein production and extraction

Production of *Ef*HTG and *Ef*XTH‐H in *Pichia pastoris* strain SMD1168H was done as described before (Simmons *et al*., 2015; Holland *et al*., 2020). Enzyme extraction from *Equisetum* followed the protocol of Fry *et al*. (2008). In brief, approximately 0.5–1.5 g of tissue was ground in extraction buffer (0.3 m succinate (Na^+^, pH 5.5), 3% (w/v) polyvinylpolypyrrolidone; 5 ml g^−1^ FW) at 0°C, and the supernatant was either stored at −80°C until processed or immediately used for assaying XET, MXE and CXE activity.

### Assaying radioactivity

Soluble ^3^H‐labelled compounds were quantified by scintillation counting in ScintiSafe 3 scintillation cocktail (Fisher Scientific, Loughborough, UK), ^3^H bound to cellulosic substrates was assayed by scintillation counting in GoldStar ‘O’ scintillation cocktail (Meridian, Chesterfield, UK).

### *In‐vitro* radiochemical assay of XET, MXE and CXE activities and their inhibition

Practical methodology for transglycanase assays is presented by Franková and Fry (2020). To test the inhibitory effects of sugar analogues and oligosaccharides on XET, MXE or CXE activity, we included 0.01–80 mm of the potential inhibitor in a reaction mixture (total volume 20 µl) containing 5 µl of filtrate from *Pichia* cultures expressing *EfHTG* or *EfXTH‐H* or *Equisetum* protein extracts, 0.1 m succinate (Na^+^, pH 5.5), 0.1% (w/v) BSA, 0.4–1.0 kBq acceptor substrate ([^3^H]XXXGol or [^3^H]XyG) and 0.5% (w/v) donor substrate (XyG or MLG for XET or MXE activity, respectively). For CXE activity, 20 mg of cellulosic substrate (insoluble donor; Whatman No. 1 filter paper that was either untreated (cellulose I) or pre‐treated with 6 m NaOH (thus cellulose II)) was soaked with 20 µl reaction mixture lacking a soluble donor substrate. Controls contained heat‐inactivated enzymes and the values obtained from these samples were subtracted from non‐mock groups, thus correcting values for unspecific [^3^H]XXXGol or [^3^H]XyG binding. Mixtures were incubated for 24 h at 20°C or at 0–60°C in a temperature chamber. XET and MXE products were dried on Whatman No. 3 paper, washed in running tap water overnight, re‐dried and quantified by scintillation counting. After the reaction had been stopped with 6 µl of 90% formic acid, cellulosic substrates were washed sequentially in 6 m NaOH for 12 h at 20°C, 6 m NaOH for 0.5 h at 100°C and running tap water overnight, and assayed for bound ^3^H. Control groups contained heat‐inactivated enzyme preparations and the signal obtained was subtracted as ‘background ^3^H’ from experimental groups if not otherwise stated.

In complementary experiments, a fluorescent dot‐blot assay testing for XET, MXE and CXE activities was used (Fry, 1997; Chormova *et al*., 2015; Franková and Fry, 2020). Whatman No. 1 filters were impregnated with XyG (for XET assays) or MLG (for MXE) by dipping into 1% (w/v) polysaccharide solution and left untreated (for CXE assays on cellulose I) or pre‐treated with 6 m NaOH (CXE on cellulose II). Dry papers were then impregnated with approximately 5 µm XXXGol‐SR, dried again, loaded with 5 µl of reaction mixture (4 µl *Equisetum* crude protein extract or *Pichia*‐produced *Ef*HTG, 0.1% BSA, 0.05 m succinate and 0–10 mm cellobiose) and incubated in darkness at 20°C between acetate stationery sheets to maintain humidity for approximately 18 h. Papers were then washed in ethanol/formic acid/water (1:1:1, v/v/v) for 1.5 h, rinsed twice with water and dried. Fluorescence emitted by bound XGO‐SR was visualised under 254 nm UV.

The pH dependency of transglucanase activities was measured in 0.1 m succinate (pH 3.2–7.7) or citrate (pH 2.1–7.6) buffers (counter‐ion Na^+^).

To test the longevity of HTG activity, we incubated MXE assay reaction mixtures (50 µl total: containing 5 µl enzyme, 2.5 kBq [^3^H]XXXGol (final concentration 78 nm XXXGol), 0.9% MLG in 0.225 m citrate (Na^+^, pH 6.3) and 0.45% chlorobutanol) for between 0.2 and 31 days at 6, 23 or 37°C. Reactions were terminated with formic acid and [^3^H]polysaccharide products were assayed by paper binding as above.

Recovery of *Ef*HTG bound to paper or cotton wool in the presence of increasing BSA concentrations was tested as follows. *Ef*HTG solution (10 µl *Pichia*‐produced *Ef*HTG in 20 µl final volume containing 0–10% (w/v) BSA) was pipetted onto 20 mg cellulose (Whatman No. 1 paper or cotton wool; 6 m NaOH‐pre‐treated or untreated), incubated for 16 h and dried at 20°C, and then the cellulose was washed in 0.05 m succinate buffer (Na^+^, pH 5.5) under gentle shaking at 20°C for 6 h. Washing solutions were collected, freeze‐dried and re‐dissolved in water. Control samples received the same treatment except that they were not applied to cellulose. The protein recovered from cellulose by washing (potentially containing *Ef*HTG) and controls that had not been in contact with cellulose were then assayed for MXE activity in a reaction mixture (total volume 20 µl) containing 10 µl of cellulose‐recovered or control *Ef*HTG, 1 kBq [^3^H]XXXGol, 1% (w/v) BSA and 0.5% (w/v) MLG.

### Testing *in‐situ* transglucanase actions in presence of cellobiose

An assay for quantifying XET, MXE and CXE action in native plant cell walls was described in detail previously (Herburger *et al*., 2020a). Briefly, mature *Equisetum* stems (approximately 250 mg) were sectioned with a razor blade and incubated with 50 kBq [^3^H]XXXGol (in 750 µl 25 mm succinate (Na^+^) + 0.1% (w/v) chlorobutanol with or without 10 mm cellobiose) for 24 h, and after the reaction had been stopped with 0.5% formic acid in 96% ethanol, specimens were washed thoroughly with ethanol (90–30%). Hemicelluloses were extracted with 6 m NaOH (4 × 24 h at 37°C) and digested with lichenase (releasing MXE‐diagnostic Glc_2_•[^3^H]XXXGol) and then with XyG endoglucanase (releasing XET‐diagnostic [^3^H]XXXGol). ^3^H in both the MXE‐ and XET‐diagnostic products was quantified by scintillation counting. The cellulosic pellet obtained after hemicellulose extraction was boiled in 6 m NaOH (1 h), re‐digested with lichenase (the additional ^3^H‐labelled material released was added to the above MXE‐diagnostic product fraction) and then treated with a series of cellulose digesting enzymes (endo‐cellulase and cellobiohydrolase; see Herburger *et al*., 2020a), releasing for quantification any [^3^H]XXXGol covalently bound to cellulose. Finally, the cellulosic pellet was treated with 2 m TFA (120°C for 1 h) and any additional released ^3^H was quantified. CXE action was recorded as the total radioactivity released from the cellulosic pellet by cellulose‐digesting enzymes and TFA treatment.

### Monitoring growth of *Equisetum* shoots in presence of cellobiose

Fifteen *E. fluviatile* explants, each consisting of a shoot (length 22.5 ± 3.8 cm, diameter at the top 1.36 ± 0.09 mm, diameter at base 2.55 ± 0.18 mm) connected to a segment of horizontal rhizome, were selected. The rhizome was cut under water giving a length of approximately 20 cm and the explants were transferred into 250‐ml beakers. Water from the pond in which the plants had been growing was filtered through a nylon mesh. Five randomly selected explants were grown indoors (21 ± 1°C, 50–60% relative air humidity, approximately 25 µmol photons m^–2^ sec^–1^) in 200 ml filtered pond water (pH 6.2, unadjusted) supplemented with 10 mm cellobiose. Five control plants were not exposed to cellobiose. The water was changed every 2½ days to avoid algal and fungal growth. After 5 days, the water was changed to sugar‐free pond water and elongation was recorded for another 10 days. After 15 days, cellobiose (10 mm) was added again and shoot growth was recorded for a further 15 days. In an additional experiment, five plants were exposed to 10 mm glucose instead of cellobiose and their growth was compared with control plants lacking any treatment over 10 days. Shoots and rhizomes were sectioned, and transglucanase action was visualised by incubation of approximately 200‐µm sections in 150 µl 25 mm succinate (Na^+^, pH 5.5) containing approximately 5 µm XXXGol‐SR and 10 or 0 mm (control) cellobiose for 2–4 h. Sections where then washed in ethanol/formic acid/water (15:1:10, v/v/v) for 10 min and in aqueous 5% (v/v) formic acid overnight to remove non‐incorporated XXXGol‐SR. After rinsing in water, sections were examined with a Leica DM2000 LED microscope equipped with a Leica DFC7000 T camera and a Leica EL6000 external light source. Incorporated SR was visualised with a GFP filter cube (excitation band pass (BP) 470/40 nm (i.e. centre wavelength 470 nm, bandwidth 40 nm), emission BP 525/50 nm). Images were taken with LAS X software and assembled in Adobe Photoshop CC. Minimal contrast adjustments were applied equally across entire image plates. Fluorescence intensity on images (*n* = 4) was quantified with ImageJ. To test solute transport from explants’ rhizome segments into their vertical shoots, we added 0.05% safranin O to pond water (200 ml, 12 h) in a separate experiment, and blotted the stem cross‐sectional area onto filter paper. Safranin O in stem cross sections (approximately 200 µm thickness) was visualised by brightfield and fluorescence microscopy. Controls lacked safranin O.

### Impregnation of filter paper with hemicelluloses and measuring transglucanase activities

Pieces (20 mg) of Whatman No. 1 filter paper (pre‐treated, or not, with 6 m NaOH, thoroughly washed in water and dried) were dipped into 0–1% (w/v) XyG, MLG or other plant polysaccharides, dried and washed in water for 4 h, removing loosely bound polysaccharides. The amount of hemicelluloses removed by washing in water (4 h) was assessed from paper weights, which were dipped in 0.5% (w/v) XyG or MLG, before and after washing and drying. Reaction mixture (enzyme, 1 kBq [^3^H]XXXGol and 0.1% (w/v) BSA, total volume 20 µl) was added to the dried, impregnated, approximately 20‐mg pieces of paper, and after 24 h incubation, the papers were washed in running tap water overnight and re‐dried, and the bound ^3^H was quantified by scintillation counting. Papers were recovered, the scintillant was removed with acetone and the papers were washed for 24 h in 6 m NaOH, which removes bound hemicelluloses; after washing in water and re‐drying, papers were assayed for firmly bound ^3^H.

### *Equisetum* transglucanases acting on *Equisetum* cell walls as donor substrates

Crude protein extracts prepared from different *Equisetum* tissues (young emerging stem, green shoot tip, green shoot middle, old blackish shoot base) were tested for XET, MXE and CXE activity on their respective pure donor substrates as described above. The *Equisetum* tissue remaining after protein extraction was washed in 75% (v/v) ethanol (thus denaturing any remaining enzymes) until the supernatant was colourless, yielding AIR. The AIR (30 mg) was soaked with 30 µl reaction mixture (20 µl enzyme extract or *Pichia*‐produced *Ef*HTG or *Ef*XTH‐H, with 1 kBq [^3^H]XXXGol and 0.05% (w/v, final concentration) BSA) and incubated at 20°C for 24 h; then, after the reaction had been stopped with formic acid, hemicelluloses were extracted with 2 ml 6 m NaOH (4 × 1 day at 37°C under constant shaking). NaOH extracts were slightly acidified with acetic acid, dialysed against tap and distilled water (3 × 1 day), freeze‐dried and digested with 250 µl of lichenase (0.06 U ml^−1^ in 25 mm citrate (Na^+^, pH 6.5), for 6 h at 20°C). Digestion products were dried, dissolved in aqueous 72% ethanol and centrifuged (2500 rpm for 10 min). The lichenase‐resistant pellet (containing XyG‐[^3^H]XXXGol; XET products) and supernatant (containing Glc_2_•[^3^H]XXXGol) were assayed for ^3^H. The remaining NaOH‐insoluble cellulosic material containing CXE products was digested by Saeman hydrolysis in H_2_SO_4_ (Saeman, 1945) and released ^3^H was quantified.

### Quantifying the effect of methanol‐stable *Equisetum* extracts on activities of *Pichia*‐produced *Ef*HTG

To test the effect of *Equisetum* extracts on *Ef*HTG’s activities (XET, MXE, CXE), which were assayed as described above, we mixed *Pichia*‐produced *Ef*HTG with *Equisetum* extracts prepared from young emerging shoots, green or black internodes or roots. These extracts had either been boiled for 5 sec, inactivating extracted enzymes, or not boiled. A reaction mixture (20 µl) contained: 5 µl enzyme solution (filtrate from *Ef*HTG‐producing *Pichia* cultures, *Equisetum* protein extracts or water (control)), 0.1 m succinate (Na^+^, pH 4.0–6.8), 0.05% (w/v) BSA, 1 kBq [^3^H]XXXGol and donor substrate (0.5% (w/v) XyG or MLG or 20 mg of cellulosic substrate (untreated or pre‐treated with 6 m NaOH at 20°C)). In an additional experiment, freeze‐dried *Equisetum* extracts were boiled (approximately 65°C) in 99.8% methanol (5 min), dialysed against water (in 12‐kDa cut‐off tubing, 2 × 1 day), dried and added (at 0.1–1% (w/v)) to assays testing for CXE activity of *Pichia*‐produced *Ef*HTG on plain or 6 m NaOH‐pre‐treated Whatman No. 1 paper.

### Statistical evaluation of the data

Experiments were usually carried out with three to six independent replicates. Data are represented as the mean and standard deviation. Statistically significant differences between groups were determined by standard *t*‐test or one‐way analysis of variance (anova) followed by Tukey’s *post hoc* test (Origin 8.5).

## CONFLICT OF INTEREST

A patent application (WO2015044209) has been filed by BASF Agricultural Solutions Belgium NV and University of Edinburgh for the use of hetero‐transglycosylase. LF, FM, AH and SCF are inventors.

## AUTHOR CONTRIBUTIONS

SCF, KH, LF, AH and FM planned and designed the study, KH performed most of the experiments, MP synthesised and assayed the [^3^H]xyloglucan, AX performed some of the inhibitor screenings, KH, SCF and LF analysed the data, KH prepared the figures and drafted the manuscript and SCF and LF edited the manuscript. All authors approved the manuscript.

## Supporting information


**Figure S1.** Extractable transglucanase activities from different *Equisetum* parts.
**Figure S2.** Effect of BSA on *Ef*HTG activities (XET, MXE, CXE).
**Figure S3.** Statistical evaluation of stimulatory effect of non‐enzymatic *Equisetum* polymers.
**Figure S4.** Safranin O uptake by hydroponically grown *Equisetum fluviatile* shoots.

**Table S1.** pH and temperature optima of the three transglucanase activities of HTG.

## Data Availability

All relevant data can be found within the manuscript and its supporting material.
