# Supplementary material for: Defining natural factors that stimulate and inhibit cellulose:xyloglucan hetero‐transglucosylation
Source: Plant J. 2021 Jan 21;105(6):1549–65. doi: 10.1111/tpj.15131 (PMC8611796; doi:10.1111/tpj.15131)
Supplement: Supplementary file 1 — Figure S1. Extractable transglucanase activities from different Equisetum parts. Figure S2. Effect of BSA on EfHTG activities (XET, MXE, CXE). Figure S3. Statistical evaluation of stimulatory effect of non‐enzymatic Equisetum polymers. Figure S4. Safranin O uptake by hydroponically grown Equisetum fluviatile shoots. [file TPJ-105-1549-s002.docx]

Supporting information

**Defining natural factors that stimulate and inhibit cellulose:xyloglucan heterotransglucosylation**

Klaus Herburger^1,3^, Lenka Franková^1^, Martina Pičmanová^1^, Anzhou Xin^1^, Frank Meulewaeter^2^, Andrew Hudson^1^, Stephen C. Fry^1^*

*^1^The Edinburgh Cell Wall Group, Institute of Molecular Plant Sciences, School of Biological Sciences, The University of Edinburgh, Edinburgh EH9 3BF, United Kingdom*

*^2^BASF, BBCC Innovation Center Gent – Trait Research, 9052 Gent (Zwijnaarde), Belgium*

*^3^Present address: Section for Plant Glycobiology, Department of Plant and Environmental Sciences, University of Copenhagen, 1871 Frederiksberg, Denmark*

*Author for correspondence: S.C. Fry (s.fry@ed.ac.uk)

(a)

MXE:XET

CXE:XET

450±110

620±80

350±10

840±60

1170±80

2460±190

*Equisetum* protein extracts

1

2

3

4

*Ef*HTG

*Ef*XTH-H

Ratios

(b)

AIR 1

AIR 2

AIR 3

AIR 4

Ratios

Extr. 1

Extr. 2

Extr. 3

Extr. 4

*Ef*HTG (*Pichia*)

*Ef*XTH-H (*Pichia*)

CXE

MXE

XET


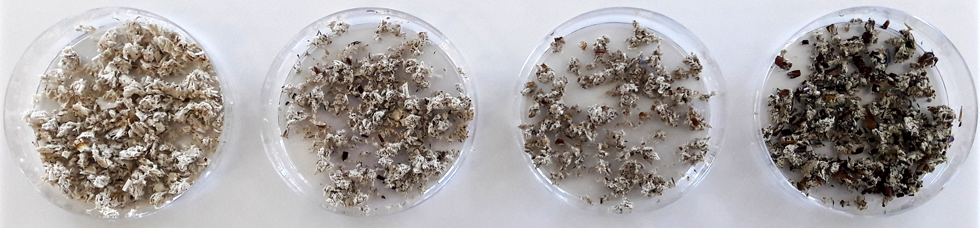


**Figure S1**

**Extractable transglucanase activities from different *Equisetum* parts.**

Acceptor substrate: [^3^H]XXXGol. (a) Donor substrate: 0.5% (w/v) XyG (for XET activity), MLG (MXE) or alkali-pretreated Whatman No. 1 paper (CXE). The *y*-axis shows MXE and CXE activity relative to XET activity (MXE:XET and CXE:XET ratio) in extracts from shoot parts of different age. Extracts 1–4 are the same as used in Figure 3. Ratios for *Pichia*-produced *Ef*HTG and *Ef*XTH-H are also shown for comparison. Absolute XET values are shown above columns in kcpm/g FW/24 h ± SD; n=3; data points shown as circles. (b) Data (XET, MXE, CXE activities) shown in Figure 3b expressed as percentages; n=3±SD.


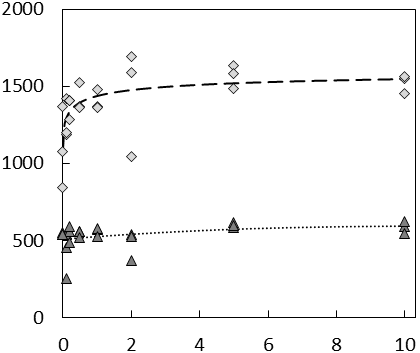


Activity (cpm/24 h)

(b)

BSA (%)

MXE

XET


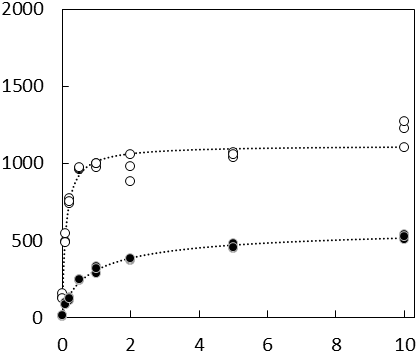


CXE activity (cpm/24 h)

(a)

BSA (%)

Cellulose I

Cellulose II

**Figure S2**

**Effect of BSA on *Ef*HTG activities (XET, MXE, CXE).**

Acceptor substrate: [^3^H]XXXGol. Enzyme: *Pichia*-produced EfHTG. (a) Absolute values (cpm/24 h) for CXE activities of experiments shown in Fig. 5a. Donor substrate: untreated (cellulose I) or NaOH pre-treated filter paper (cellulose II); n=3; data points shown as circles. (b) Absolute values (cpm/24 h) for XET and MXE activities of experiments shown in Fig. 5b. Donor substrates: XyG or MLG; n=3; data points shown as rhombuses (MXE) and triangles (XET).


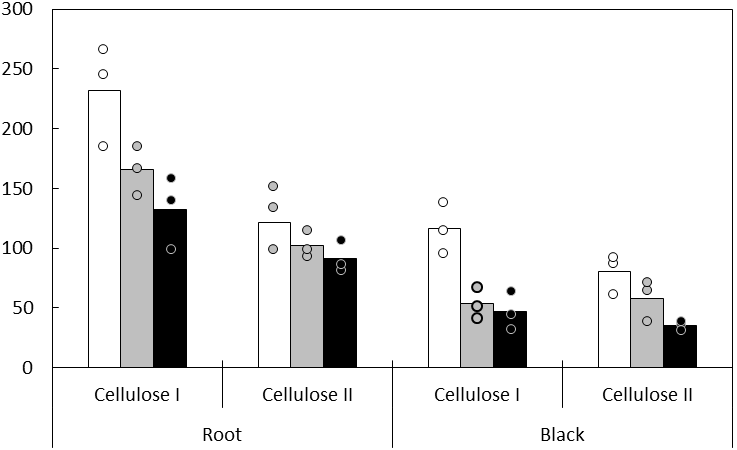


Stimulation of CXE activity by extract boiled in methanol (%)

a

b

b

A

AB

B

a

b

b

A

AB

B

pH 4

pH 5.5

pH 6.8

**Figure S3**

**Statistical evaluation of stimulatory effect of non-enzymic *Equisetum* polymers.**

Stimulation of CXE activity of *Pichia*-produced *Ef*HTG in the presence of activity-stimulating *Equisetum* crude extracts boiled in methanol as shown in Figure 4c. Extracts: Root, from roots; Black, from blackish shoot base. Stimulation was determined when cellulose I or cellulose II was used as CXE donor substrate and at pH 4.0, 5.5 or 6.8  . Statistically significant differences between stimulations at different pH values are indicated by small letters (cellulose I, root), capital letters (cellulose I, black), underlined small letters (cellulose II, root) and underlined capital letters (cellulose II, black). They were determined by one-way ANOVA followed by Tukey’s post hoc test; n=3; data points shown as circles, p<0.05.

(a)

(b)

Top


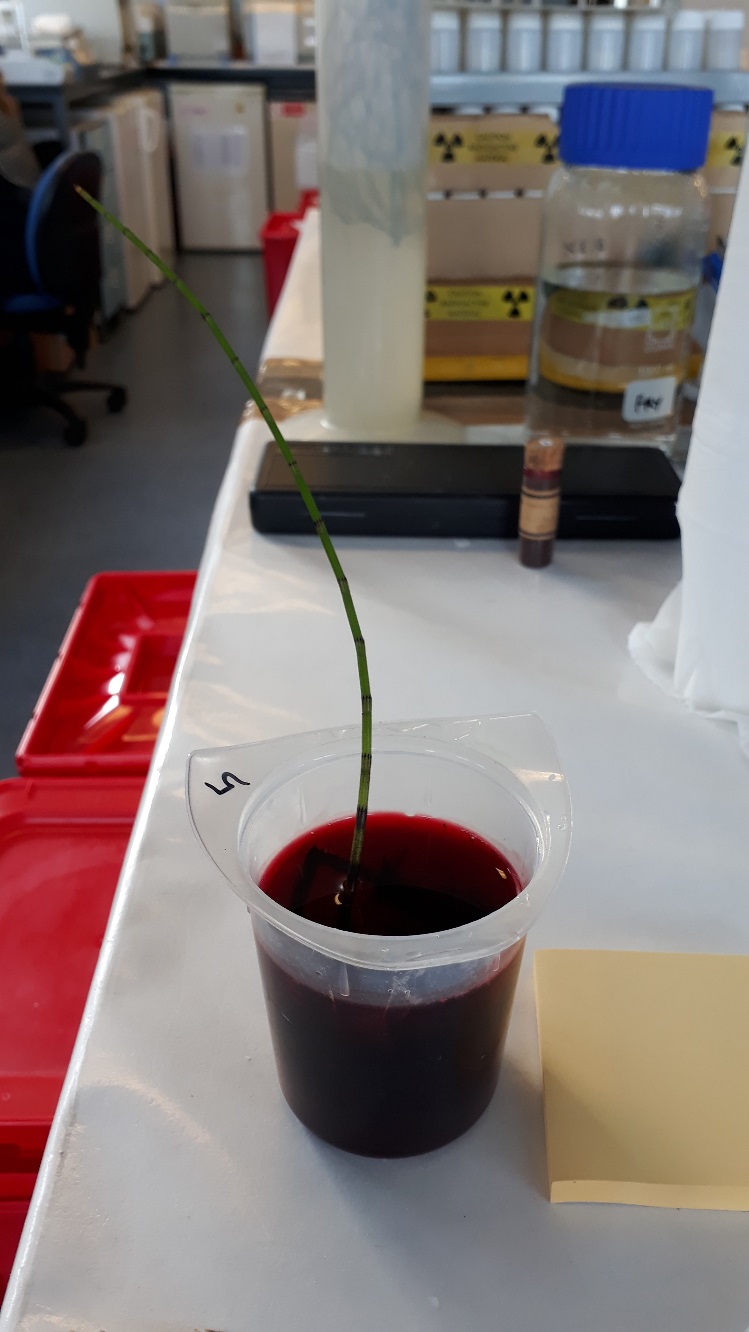


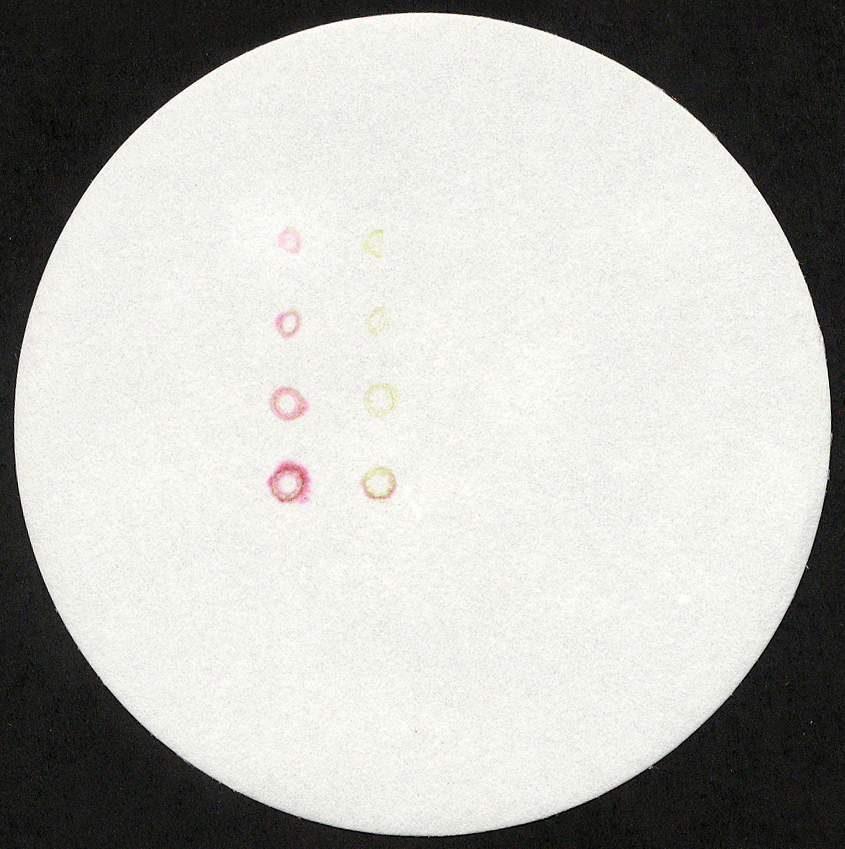


Top

8 cm from top

16 cm from top

Base

**12 h incubation**

1 cm

**-Safranin**

**+Safranin**

8 cm from top

16 cm

from top

Base

(c)


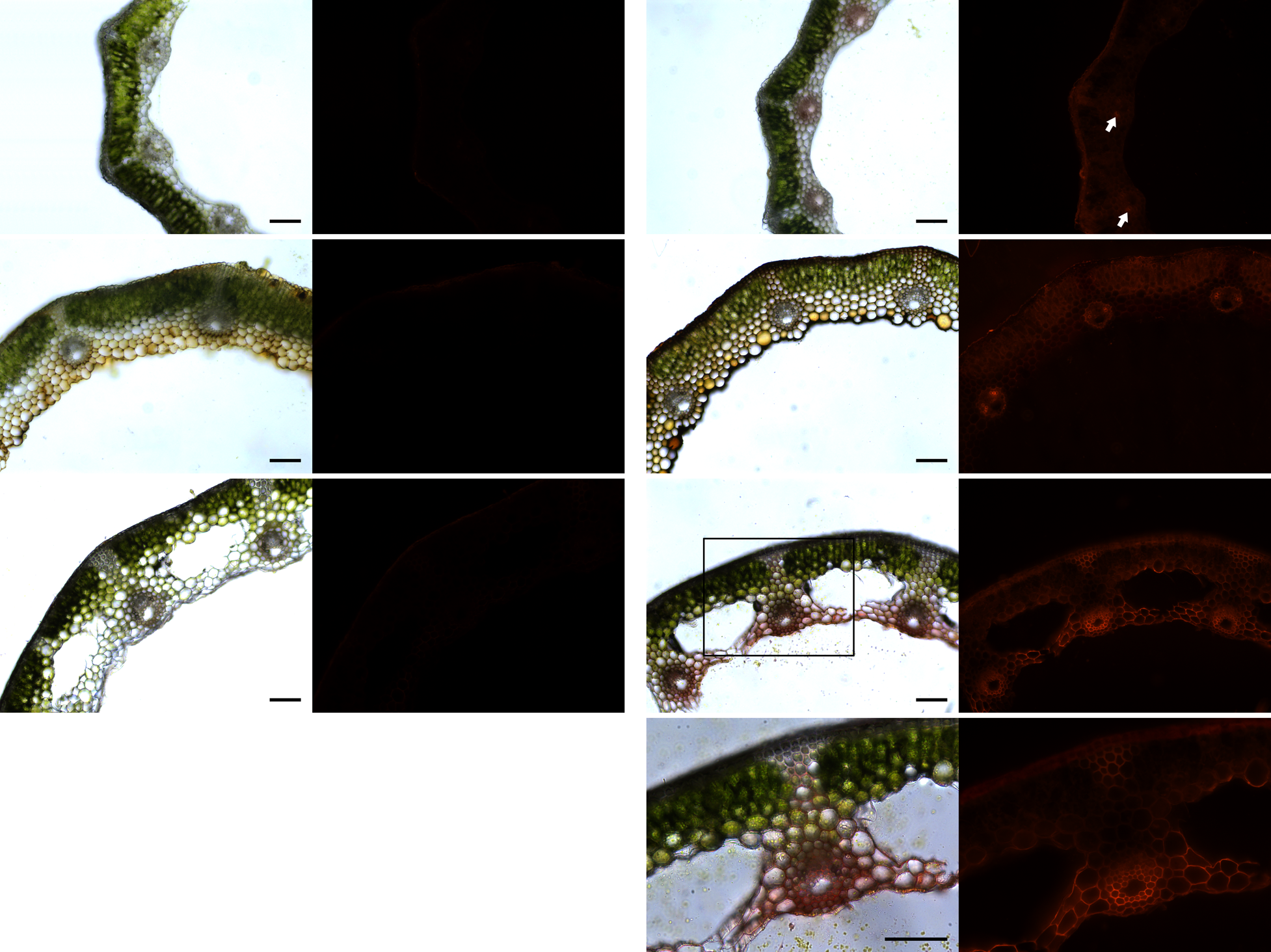


Top

Top

Middle

Middle

Base

Base

**Figure S4**

**Safranin uptake by hydroponically grown**

***Equisetum fluviatile* shoots.**

For legend, see next page

**Figure S4**

**Safranin uptake by hydroponically grown *Equisetum fluviatile* shoots.**

(a) *Equisetum* shoot connected to a rhizome segment, exposed to 0.05% safranin and ~25 µmol photons m^–2^ s^–1^ for 12 h. Sites for cross-sectioning in (b) are marked. (b) Filter-paper blotting of segments from the *Equisetum* shoot shown in (a) and a similar shoot incubated in the absence of safranin. Safranin distributed in tissues can be detected from the shoot base to the top internode (red blots). (c) Microscopic detection of safranin distribution in tissues (bright field; red colour) and corresponding fluorescence images (red fluorescence). Right: Safranin occurs in vascular bundles (arrows: metaxylem), adjacent parenchyma, chlorenchyma, epidermis and stem-base sclerenchyma. Inset: Vascular bundle flanked by vallecular canals. Left: Autofluorescence of sections from shoots not exposed to safranin is barely detectable. Scale bar 250 µm.
