# Supplementary material for: Defining natural factors that stimulate and inhibit cellulose:xyloglucan hetero‐transglucosylation
Source: Plant J. 2021 Jan 21;105(6):1549–65. doi: 10.1111/tpj.15131 (PMC8611796; doi:10.1111/tpj.15131)
Supplement: Supplementary file 2 — Table S1. pH and temperature optima of the three transglucanase activities of HTG. [file TPJ-105-1549-s001.docx]

Supporting information

**Defining natural factors that stimulate and inhibit cellulose:xyloglucan heterotransglucosylation**

Klaus Herburger^1,3^, Lenka Franková^1^, Martina Pičmanová^1^, Anzhou Xin^1^, Frank Meulewaeter^2^, Andrew Hudson^1^, Stephen C. Fry^1^*

*^1^The Edinburgh Cell Wall Group, Institute of Molecular Plant Sciences, School of Biological Sciences, The University of Edinburgh, Edinburgh EH9 3BF, United Kingdom*

*^2^BASF, BBCC Innovation Center Gent – Trait Research, 9052 Gent (Zwijnaarde), Belgium*

*^3^Present address: Section for Plant Glycobiology, Department of Plant and Environmental Sciences, University of Copenhagen, 1871 Frederiksberg, Denmark*

*Author for correspondence: S.C. Fry (s.fry@ed.ac.uk)

**Table S1**

**pH and temperature optima of the three transglucanase activities of HTG.**

|  | **pH optimum** | | **Temperature** |
| --- | --- | --- | --- |
| **Activity** | **succinate** | **citrate** | **optimum (°C)** |
| **XET** | **5.64±0.13^a^** | **5.73±0.19^A^** | **25.1±1.3^a^** |
| **MXE** | **5.64±0.16^a^** | **5.64±0.18^A^** | **23.8±1.5^a^** |
| **CXE** | **5.63±0.06^a^** | **5.68±0.14^A^** | **33.7±2.1^b^** |

Values were derived from Fig. 1a (pH) and Fig. 1b (temperature), n=3±SD. Significant differences between the activities are indicated by small letters (pH optima succinate), capital letters (pH optima citrate) and underlined letters (temperature optima), as determined by one-way ANOVA (p < 0.05) followed by Tukey’s post hoc test.
